# Surf4, cargo trafficking, lipid metabolism, and therapeutic implications

**DOI:** 10.1093/jmcb/mjac063

**Published:** 2022-11-29

**Authors:** Yishi Shen, Hong-Mei Gu, Shucun Qin, Da-Wei Zhang

**Affiliations:** Group on the Molecular and Cell Biology of Lipids and Department of Pediatrics, Faculty of Medicine and Dentistry, University of Alberta, Edmonton, AB T6R 2G3, Canada; Group on the Molecular and Cell Biology of Lipids and Department of Pediatrics, Faculty of Medicine and Dentistry, University of Alberta, Edmonton, AB T6R 2G3, Canada; Institute of Atherosclerosis in Shandong First Medical University (Shandong Academy of Medical Sciences), Taian 271016, China; Group on the Molecular and Cell Biology of Lipids and Department of Pediatrics, Faculty of Medicine and Dentistry, University of Alberta, Edmonton, AB T6R 2G3, Canada

**Keywords:** VLDL secretion, cargo receptor, PCSK9, lipid metabolism, atherosclerosis, trafficking

## Abstract

Surfeit 4 is a polytopic transmembrane protein that primarily resides in the endoplasmic reticulum (ER) membrane. It is ubiquitously expressed and functions as a cargo receptor, mediating cargo transport from the ER to the Golgi apparatus via the canonical coat protein complex II (COPII)-coated vesicles or specific vesicles. It also participates in ER–Golgi protein trafficking through a tubular network. Meanwhile, it facilitates retrograde transportation of cargos from the Golgi apparatus to the ER through COPI-coated vesicles. Surf4 can selectively mediate export of diverse cargos, such as PCSK9 very low-density lipoprotein (VLDL), progranulin, **α**1-antitrypsin, STING, proinsulin, and erythropoietin. It has been implicated in facilitating VLDL secretion, promoting cell proliferation and migration, and increasing replication of positive-strand RNA viruses. Therefore, Surf4 plays a crucial role in various physiological and pathophysiological processes and emerges as a promising therapeutic target. However, the molecular mechanisms by which Surf4 selectively sorts diverse cargos for ER–Golgi protein trafficking remain elusive. Here, we summarize the most recent advances in Surf4, focusing on its role in lipid metabolism.

## Introduction

Eukaryotic cells have a complex endomembrane system that forms different compartments within the cell, such as the endoplasmic reticulum (ER), Golgi apparatus, endosomes, and lysosomes. Materials are exchanged among these compartments using different mechanisms. Thousands of proteins are synthesized in the ER, then sorted and transported to the Golgi apparatus for secretion or delivery to specific destinations within cells (Otte and Barlowe, 2004; Szul and Sztul, 2011; Barlowe and Helenius, 2016; Gomez-Navarro and Miller, 2016; Peotter et al., 2019; Shomron et al., 2021; Yan et al., 2022). Several pathways coexist to transport newly synthesized cargos out of the ER, such as the coat protein complex II (COPII) vesicle and a tubular trafficking network. Currently, the most widely studied mechanism is the canonical COPII vesicle, where ER-to-Golgi trafficking starts at the ER exit sites (ERESs), and cargo receptors or cargos then recruit COPII to generate protein transport vesicles for delivery to the Golgi apparatus (Tiwari and Siddiqi, 2012; Gomez-Navarro and Miller, 2016; Peotter et al., 2019). Bulk flow that does not require cargo sorting also exists in this process since a transport vesicle can incorporate lipids and soluble proteins by default. Several studies have demonstrated that the bulk flow rate is fast enough to support the rate of protein transportation in the secretory pathway (Wieland et al., 1987; Bethune et al., 2006; Spang, 2013; Barlowe and Helenius, 2016; Gomez-Navarro and Miller, 2016; Peotter et al., 2019). However, whether bulk flow is a general mechanism regulating ER export of soluble cargo proteins remains to be further investigated. On the other hand, coat protein complex I (COPI) operates retrograde transportation from the Golgi apparatus to the ER to maintain proper localization and homeostasis of ER- and Golgi-resident proteins (Bethune et al., 2006; Spang, 2013; Arakel and Schwappach, 2018). In addition, emerging evidence shows that ERES is composed of a complex, intertwined tubular network. In this intricate network, COPII and COPI govern cargo anterograde and retrograde transportation, respectively. Pearly tubular vesicles containing proteins transported to the Golgi apparatus are also observed alongside the microtubules extending out of the ERESs (Martinez-Menarguez et al., 1999; Weigel et al., 2021; Yan et al., 2022). This endomembrane network is precisely orchestrated to maintain precise protein transportation and homeostasis of different compositions in distinct organelles, ensuring normal physiological functions of cells and tissues. Recently, emerging evidence supports a crucial role of Surfeit 4 (Surf4) in mediating secretion of diverse cargos, such as proprotein convertase subtilisin/kexin type 9 (PCSK9), very low-density lipoprotein (VLDL), progranulin, α1-antitrypsin (α1-AT), STING, proinsulin, and erythropoietin (EPO). Here, we reviewed the physiological functions of Surf4, with particular emphasis on recent advances in the role of Surf4 in lipid metabolism, which may pave the way for the development of potential novel clinical interventions for dyslipidaemia and other related human diseases.

## Discovery of Surfeit locus genes

The mouse Surfeit locus was first reported by Williams et al. in 1986. They observed that Mes-1, a murine enhancer element, is located within 15–73 base pairs between the heterogenous 5′ ends of two different genes, Surf1 and Surf2. They then found that the 3′ end of the third transcription, Surf3, is located 70 bp from the 3′ end of Surf1, and the 3′ end of the fourth transcription, Surf4, overlaps with the 3′ end of Surf2 by 133 bp (Williams and Fried, 1986a, b; Williams et al., 1988). The same lab identified Surf5 and Surf6 in 1990. The 5′ end of Surf6 is located within a CpG-rich island about 8 kilobases from a CpG-rich island containing the 5′ end of Surf3, and Surf5 resides between Surf3 and Surf6. The surfeit cluster contains all Surf1–Surf6 genes flanked by a CpG-rich island (Huxley and Fried, 1990). Surf1 encodes an integral membrane protein, and mutations in Surf1 cause Leigh syndrome, a severe neurological disorder characterized by progressive loss of mental and movement abilities (Mashkevich et al., 1997; Da-Re et al., 2014). Surf3 encodes a ribosomal protein called L7a, which promotes tumorigenesis, such as in breast cancer and osteosarcoma (Giallongo et al., 1989; Zhu et al., 2001; Zheng et al., 2009). Surf5, now named MED22, has three different transcripts, Surf-5a, Surf-5b, and Surf-5c, due to alternative splicing (Garson et al., 1995, 1996; Angiolillo et al., 2002). Disruption of MED22 has been reported to be associated with the formation of intracellular vacuoles in podocytes, and MED22 is required to maintain podocyte health (Rodriguez et al., 2020). Surf6 is located in the nucleolus, participates in rRNA processing during ribosome biogenesis, and may promote tumorigenesis (Moraleva et al., 2017).

## Surf4, a highly conserved cargo receptor

**Figure 1:**
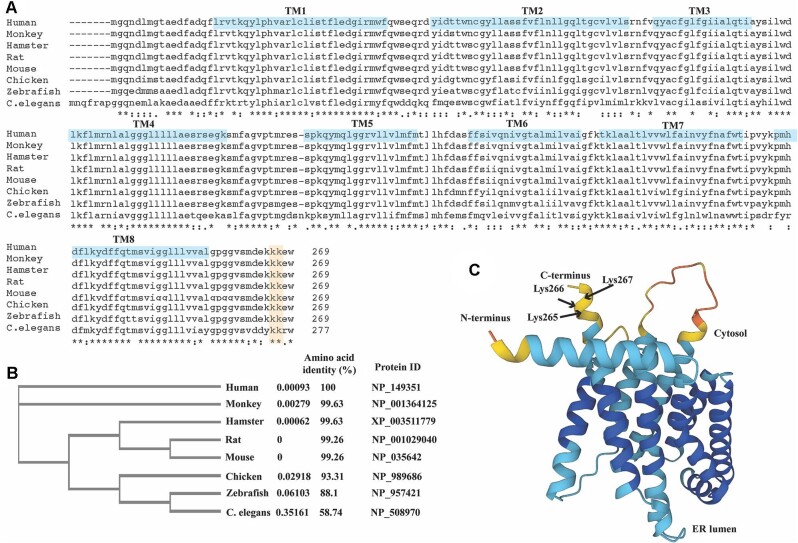
Structure of Surf4. (**A**) Sequence alignment in CLUSTAL format. The alignment of Surf4 among different species, including human, monkey, hamster, rat, mouse, chicken, zebrafish, and *C. elegans*. The C-terminal di-lysine ER-localization motif is highlighted in light orange. Eight putative transmembrane (TM) domains predicted by AlphaFold are highlighted in light blue. (**B**) Phylogenetic tree of Surf4 from different species. The branch length is shown in the cladogram. The number next to each species indicates the actual branch length. (**C**) The structure of Surf4 predicted by AlphaFold (Q15260). Surf4 is predicted to have eight putative transmembrane domains, a cytosolic exposed N-terminal domain, and a C-terminal domain. Arrows indicate the three C-terminal lysine residues. Different colors indicate different confidences of structure prediction: very high confidence (dark blue); confident (light blue); low confidence (yellow); very low confidence (red). The alignment was performed using CLUSTAL O (1.2.4) (Madeira et al., 2022).

Surf4 is highly conserved across species. Human Surf4 shares 99% amino acid identity with the monkey, hamster, rat, and mouse protein, and 93%, 88%, and 58% amino acid identity with the chicken, zebrafish, and *Caenorhabditis elegans* homologues, respectively (Reeves and Fried, 1995; Figure 1A and B). Surf4 is a mammalian homology of Erv29p, a cargo receptor in yeast; both share ∼30% amino acid identity (Caldwell et al., 2001). Surf4 consists of 269 amino acids with a molecular weight of 30 kDa and is primarily localized in the ER. It is a polytopic transmembrane protein with eight putative transmembrane α-helices and a cytosolic exposed N- and C-terminal domain (Reeves and Fried, 1995; Jumper et al., 2021). The N-terminal domain begins with a 21 amino acid-α-helix, followed by a short 3-amino acid loop connecting the first transmembrane α-helix. The C-terminal domain has a short α-helix of eight amino acid residues connected to the last transmembrane α-helix by a 6-amino acid loop. A di-lysine ER localization motif is located near the end of the C-terminal domain (Figure 1C; Andersson et al., 1999; Jumper et al., 2021). Although Surf4 has been discovered for over three decades, its physiological function has not been well studied until the last 10 years.

### Surf4 and cargo trafficking

In 2008, Mitrovic and his colleagues found that Surf4 was mainly localized within the ERGIC-53-associated structure with some overlapping with early Golgi domains in Hela cells. They also reported that Surf4 appeared to cycle between the ER and the Golgi apparatus, as replacement of the three lysine residues in the C-terminal di-lysine ER localization motif with a serine residue caused accumulation of Surf4 in the Golgi apparatus (Mitrovic et al., 2008). Later, Yin et al. (2018) observed that Surf4 could recognize and bind an amino-terminal tripeptide motif in secretory proteins, such as dentin sialophosphoprotein (DSPP), amelogenin, X-linked (AMELX), and growth hormone (GH). The consensus motif of the amino-terminal tripeptide, named the ER-ESCAPE motif, consists of bulky hydrophobic–proline–bulky hydrophobic amino acid (Φ–P–Φ) and is exposed after removal of the signal peptide in cargos. The bulky hydrophobic amino acid residues include Ile, Leu, Val, and Phe. Removal of Pro in the middle of the motif or the presence of an acidic amino acid residue in the motif significantly reduces binding of these proteins to Surf4. Upon binding to the ER-ESCAPE motif in the cargo, Surf4 is proposed to undergo a conformational change in the transmembrane domains, enabling it to interact with COPII proteins, such as Sec24, which subsequently facilitates the incorporation of the cargo into the COPII vesicle for ER export (Yin et al., 2018). Many properties of the extracellular environment of higher eukaryotes, such as neutral pH and a calcium concentration of ∼1 mM, are similar to those within the ER lumen, which allows the premature assembly of monomers to form large protein complexes (Vitale and Denecke, 1999). To prevent premature aggregation, proteins most likely to aggregate in the ER lumen, such as DSPP, GH, AMELX, have a strong ER-ESCAPE motif to facilitate their secretion and keep their concentration in the ER lumen low, while less susceptible cargos have a weaker ER-ESCAPE motif (Yin et al., 2018).

Surf4 has been implicated in ER export of diverse cargos, such as EPO, α1AT, and proinsulin (Lin et al., 2020; Ordonez et al., 2021; Saegusa et al., 2022). EPO is mainly secreted into the blood by interstitial cells in the peritubular capillary bed of the renal cortex. EPO binds to the erythropoietin receptor on erythroid precursors to promote cell proliferation and differentiation. Lack of circulating EPO, such as in patients with chronic kidney disease, can lead to severe anaemia (Maxwell et al., 1997; Jelkmann, 2016; Shih et al., 2018; Newton and Sathyanesan, 2021). Deficiency of Surf4 in HEK293T cells caused ER accumulation and extracellular depletion of EPO, whereas Surf4 overexpression in mice increased serum EPO levels, indicating the important role of Surf4 in EPO secretion (Lin et al., 2020). α1AT is mainly produced and secreted as a monomer or polymer by hepatocytes. Circulating α1AT monomer functions as a serine protease inhibitor to suppress the activity of neutrophil elastase, while α1AT polymer acts as a neutrophil chemo-attractant to stimulate inflammation (Gooptu and Lomas, 2008). Mutations that impair α1AT secretion result in accumulation of α1AT polymers in the ER of hepatocytes, increasing the risk of neonatal hepatitis and hepatocellular carcinoma (Wu et al., 1994). Ordonez et al. (2021) reported that Surf4 mediated ER–Golgi transport of both α1AT monomer and polymer in CHO cells, with a preference for the polymer. In addition, Saegusa et al. (2022) reported that Surf4 directly interacted with proinsulin and mediated the transport of proinsulin from the ER to the Golgi apparatus in cultured rat pancreatic beta cells. Silencing of Surf4 reduced insulin secretion and caused ER retention of proinsulin. Furthermore, Surf4 is required for the trafficking of progranulin to lysosomes. Progranulin can promote wound healing, stimulate tumor growth and migration, modulate the immune response, and prevent neurodegeneration. Insufficiency in progranulin also causes frontotemporal dementia and increases the risk of Alzheimer's and Parkinson's disease (Nicholson et al., 2016; Chitramuthu et al., 2017; Zhou et al., 2017; Terryn et al., 2021). Devireddy and Ferguson (2022) reported that newly synthesized progranulin and prosaposin formed a complex in the ER lumen, then prosaposin bound to Surf4 for the export of the complex from the ER.

**Figure 2:**
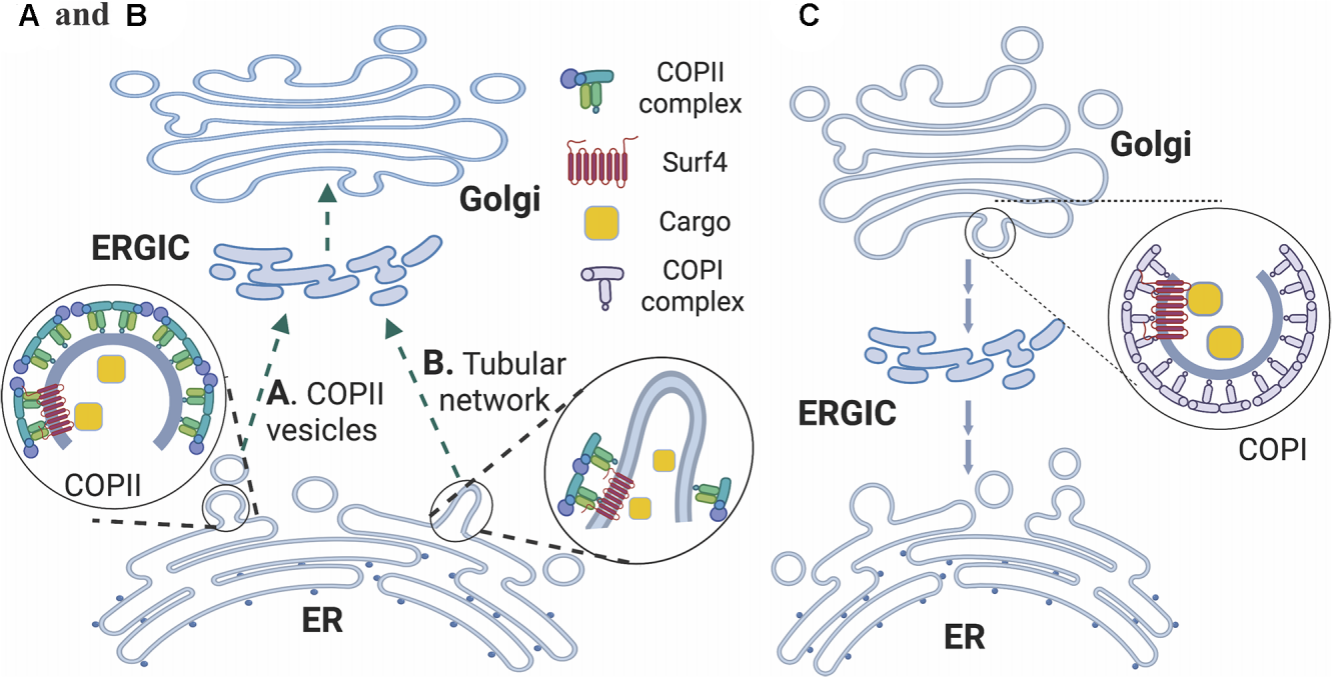
Surf4 and cargo transportation between the ER and the Golgi apparatus. (**A**) The canonical COPII vesicle-mediated ER-to-Golgi cargo transport. Newly synthesized cargoes in the ER lumen are sorted by Surf4 into the canonical COPII vesicle. Sec23/24 coat protein forms the inner layer and recruits Sec13/31 forming the second coat layer to complete COPII vesicle assembling. (**B**) The tubular network-mediated ER-to-Golgi cargo transport. A network of tubules is formed at ERES. COPII components present in the neck of the tubules to concentrate secretory proteins and then promote their entry into the tubular carriers, but do not present in the tubules. Surf4 may recognize cargos and mediate their incorporation into the tubular transport carrier. (**C**) The COPI vesicle-mediated Golgi-to-ER cargo transport. Surf4 interacts with α-COP in the COPI complex and facilitates the incorporation of cargos into the COPI vesicle for the retrograde transport from the Golgi apparatus to the ER. This figure is created with BioRender.com.

Notably, progranulin contains, whereas EPO and proinsulin do not have the N-terminal ER-ESCAPE motif, and α1AT even has an unfavorable N-terminal ER-ESCAPE motif (EDPQ). These findings indicate that the ER-ESCAPE motif is required for the ER export of some but not all Surf4’s substrates. Surf4 is located in the ERES and is involved in the formation of COPII-positive ERES (Saegusa et al., 2018). It may interact with the ER-export motif on cargos and Sec24 in COPII to mediate ER–Golgi transport of cargos via the classical COPII vesicles (Figure 2A). In addition, Surf4 is involved in the formation of the tubular network of ER–Golgi protein transport, another important carrier for the ER export of secretory proteins. ERES induces the formation of a network of tubules that, unlike COPII vesicles, contain secretory cargoes, but do not have COPII components. COPII components are present only in the neck of the tubules to concentrate secretory proteins and then promote their entry into the tubular carriers (Shomron et al., 2021; Weigel et al., 2021). Yan et al. (2022) reported that Surf4 induced the formation of a highly elongated tubular ER–Golgi intermediate compartment (t-ERGIC), accelerating ER-to-Golgi transport of soluble cargoes (Figure 2B). It will be of interest to determine whether and how Surf4 decides which of the two different ER-to-Golgi transport systems is used for ER export of different cargos.

In addition to its important role in regulating anterograde trafficking of cargos, Surf4 is critical for intracellular retrograde trafficking (Figure 2C). The C-terminal cytosolic region of Surf4 has a triple-lysine COPI sorting motif that can interact with COPI subunit α-COP to transport Surf4 back to the ER (Jackson et al., 2012). Surf4 with a mutant COPI sorting motif, in which the C-terminal three lysine residues are substituted with an alanine residue, exhibits reduced binding to α-COP and is trapped in the Golgi apparatus instead of being returned to the ER. Furthermore, deficiency of Surf4 impairs the interaction between STING and α-COP, leading to Golgi accumulation of STING and subsequent activation of the STING signaling pathway (Mukai et al., 2021). This may shed light on the pathogenesis of COPA syndrome, a rare immune disorder characterized by high titer antibodies and inflammatory arthritis (Deng et al., 2020b). Therefore, Surf4 acts as a cargo receptor for STING in the COPI-mediated retrograde trafficking and may be a potential target for the diagnosis and treatment of COPA syndrome (Deng et al., 2020b; Mukai et al., 2021). Interestingly, it has been reported that the tubular ER-to-Golgi complex is positive for COPI and acquires COPI while it moves toward the Golgi apparatus, suggesting initiation of retrograde trafficking back to the ER (Phuyal and Farhan, 2021). However, we cannot exclude the possibility that COPI may have a potential role in anterograde ER-to-Golgi transport in the tubular network (Shomron et al., 2021; Weigel et al., 2021). Nevertheless, these findings indicate that Surf4 has a broad substrate spectrum, traffics bidirectionally, and mediates both vesicle and tubular anterograde ER-to-Golgi transport.

### Surf4, lipoprotein metabolism, and atherosclerosis

Atherosclerotic cardiovascular disease, one of the leading causes of morbidity and mortality worldwide, is a chronic inflammatory disease with the buildup of atherosclerotic plaques in the arterial wall. High plasma low-density lipoprotein cholesterol (LDL-C) levels are a well-known risk factor for the development and progression of atherosclerosis (Goldstein and Brown, 2009). Plasma LDL-C levels are determined by its production and clearance. Circulating LDL is produced by catabolism of VLDL and primarily cleared by LDL receptor (LDLR). In addition, PCSK9 plays a central role in regulating plasma LDL-C homeostasis through promoting LDLR degradation (Lagace, 2014; Guo et al., 2020). Recently, it has been reported that Surf4 can facilitate secretion of PCSK9 overexpressed in HEK293 cells and mediate VLDL secretion from hepatocytes (Emmer et al., 2018; Shen et al., 2020; Wang et al., 2021a, b; Shen et al., 2022). Furthermore, a SNP (rs3758348) within the first intron of human Surf4 is significantly associated with a reduction in plasma total cholesterol and LDL-C levels (Wang et al., 2021b). These reports indicate a critical role of Surf4 in regulating lipid metabolism.

#### Surf4 and PCSK9

PCSK9 is a member of the subtilisin-like serine protease family, which includes 7 basic amino acid-specific proprotein convertases and two members (site-1 protease and PCSK9) that cleave at the carboxyl terminus of nonbasic residues in their substrates (Zhou et al., 1999; Seidah et al., 2008; Gu and Zhang, 2015). PCSK9 is a secretory glycoprotein of 692 amino acids. In 2003, Abifadel et al. (2003) reported that gain-of-function mutations in the *PCSK9* gene cause severe hypercholesterolemia. In contrast, loss-of-function mutations in PCSK9 result in a reduction in plasma LDL-C levels and are associated with reduced risk of coronary artery disease (Cohen et al., 2006). The role of PCSK9 in homeostatic control of plasma LDL-C levels is mainly dependent on its ability to promote LDLR degradation, thereby preventing LDL clearance (Rashid et al., 2005; Lagace et al., 2006; Qian et al., 2007; Zhang et al., 2007; Gu et al., 2013). In addition, inhibition of PCSK9 can reduce plasma levels of lipoprotein(a) and postprandial triglycerides (Le May et al., 2009; Romagnuolo et al., 2015; O'Donoghue et al., 2019).

Humanized monoclonal antibodies against PCSK9 have been developed with impressive clinical outcomes, reducing plasma LDL-C levels by ∼60% and a further ∼15% reduction in cardiovascular events when combined with statins (Fernandez-Ruiz, 2019; Sabatine, 2019; Szarek et al., 2019; Xia et al., 2021). However, the treatment is expensive because it requires injection of a large amount of anti-PCSK9 antibodies. PCSK9 siRNA (Inclisiran) is also effective in reducing LDL-C levels by ∼50%, but requires only two injections per year (Ray et al., 2020; Wright et al., 2021), and may therefore be more affordable. However, it remains a financial burden as a primary prevention measure for all eligible patients. In addition, siRNAs, especially at high doses, can exhibit miRNA-like off-target activity (Jackson et al., 2003) and trigger an innate immune response (Pecot et al., 2011). Patients with Inclisiran treatment show an increase in mild-to-moderate bronchitis (4.3% vs. 0.7% for Inclisiran and placebo, respectively) (Wright et al., 2021). Therefore, potential long-term side effects of using siRNAs as a lifelong primary prevention strategy still need to be assessed, and a new strategy to inhibit PCSK9 as an affordable and safe primary prevention measure for all eligible patients is in urgent need.

PCSK9 is synthesized as a zymogen and undergoes autocatalytic cleavage in the ER, which is required for PCSK9 maturation and secretion (Benjannet et al., 2004). PCSK9 is expressed in various tissues, such as the liver, kidneys, and intestine. However, circulating PCSK9 is primarily secreted by hepatocytes and has a very short half-life of ∼5 min (Grefhorst et al., 2008; Zaid et al., 2008). Furthermore, subjects carrying loss-of-function mutations in PCSK9 that impair PCSK9 secretion and reduce plasma levels of PCSK9 and LDL-C do not show notable healthy problems (Zhao et al., 2006). Overexpression of mutant PCSK9 retained in the ER also does not cause unfolded protein response or ER stress (Lebeau et al., 2018). Therefore, inhibition of PCSK9 secretion represents a promising strategy for reducing plasma PCSK9 levels. However, the mechanism of PCSK9 secretion is not fully understood. We and others have reported that SEC24, an adaptor protein of COPII vesicles, was required for PCSK9 secretion (Chen et al., 2013; Deng et al., 2020a). Furthermore, knockdown of SEC24A, SEC24B, or SEC24C significantly reduced secretion of the wild-type PCSK9 but not mutant PCSK9 without the C-terminal histidine-rich domain, suggesting the requirement of the C-terminal domain for SEC24-mediated PCSK9 secretion (Deng et al., 2020a). SEC24, an essential subunit of COPII vesicles, is localized in the cytosol and forms a complex with SAR1 and SEC23 to constitute the inner layer of the COPII vesicles. The cargo-binding sites on SEC24 recognize and directly interact with the cytosolic ER export signals present in transmembrane proteins or cargo receptors to selectively recruit cargos into COPII vesicles for ER–Golgi transport. Cargo receptors are transmembrane proteins with an ER lumen domain that binds ER luminal cargos and a cytosolic domain that interacts with COPII components, thereby sorting cargo into COPII vesicles (Wendeler et al., 2007; Mancias and Goldberg, 2008; Miller and Schekman, 2013; Chatterjee et al., 2021; Shomron et al., 2021). PCSK9 is present in the ER lumen and thus needs a cargo receptor to bridge its interaction with cytosolic SEC24.

Recently, Emmer et al. (2018) combined proximity-dependent biotinylation with CRISPR-mediated functional genomic screening to identify Surf4 as a cargo receptor for facilitating PCSK9 secretion in HEK293 cells. They found that inactivation of Surf4 led to intracellular accumulation of PCSK9 overexpressed in HEK293T cells. However, lack of Surf4 only partially reduced PCSK9 secretion. The authors proposed that Surf4 actively recruited PCSK9 into COPII vesicles, and the residual Surf4-independent secretion was possibly due to bulk flow or alternative ER cargo receptors (Emmer et al., 2018). On the other hand, we found that knockdown of Surf4 expression in two cultured human hepatoma-derived cell lines, Huh7 and HepG2 cells, did not impair endogenous PCSK9 secretion. Conversely, silencing Surf4 increased PCSK9 expression at the transcriptional level (Shen et al., 2020). Furthermore, knockout or knockdown of hepatic Surf4 in mice did not affect PCSK9 levels in the blood and liver, indicating that Surf4 is not required for endogenous PCSK9 secretion from hepatocytes *in vitro* and *in vivo* (Wang et al., 2021a). Interestingly, Wolf et al. (2020) reported that Surf4 facilitated PCSK9 secretion from cardiomyocytes, and PCSK9 then impaired cardiac function in an autocrine manner. They silenced Surf4 expression in ventricular cardiomyocytes isolated from adult Wistar rats and found that deficiency of Surf4 reduced the levels of PCSK9 released from cardiomyocytes and protected heart function. These findings suggest that Surf4 may facilitate PCSK9 secretion in a cell type-dependent manner. Emmer et al. (2018) investigated secretion of PCSK9 overexpressed in HEK293T cells that do not express endogenous PCSK9. Wolf et al. (2020) studied secretion of endogenous PCSK9 from cardiomyocytes that express PCSK9 at very low levels. On the other hand, we used *in vitro* and *in vivo* models to study secretion of PCSK9 in hepatocytes, which contributes to the majority of circulating PCSK9. Nevertheless, given the critical role of PCSK9 in regulating plasma LDL-C levels, further studies are required to elucidate the exact role of Surf4 in PCSK9 secretion and the machinery system controlling PCSK9 secretion *in vivo*.

#### Surf4 and VLDL

**Figure 3:**
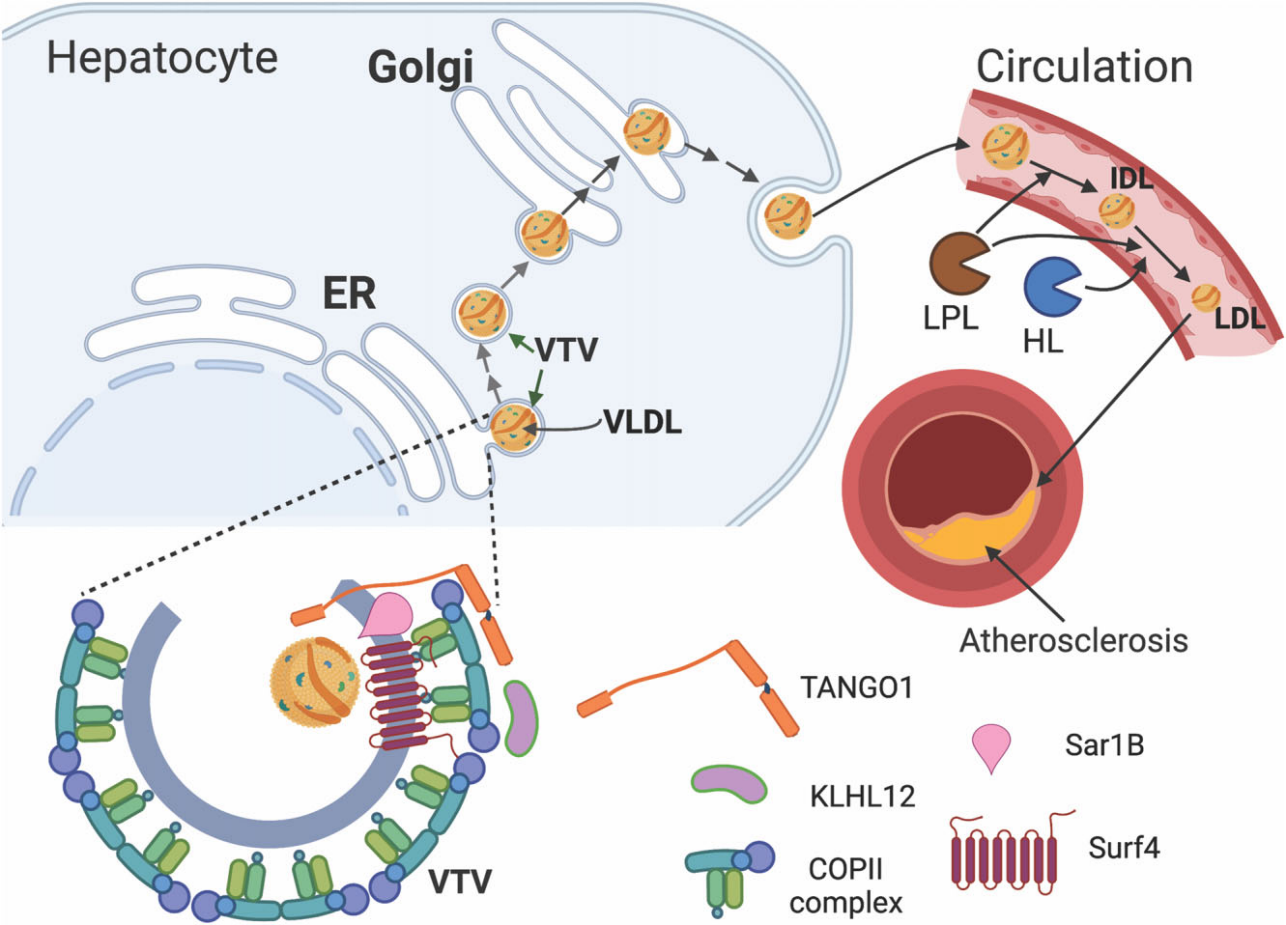
VLDL secretion and metabolism. The transport of VLDL from the ER to the Golgi apparatus is mediated by VTV, which is formed in the ERES and ranges from 100 nm to 120 nm in diameter. The biogenesis of VTV requires several factors, such as COPII components, KLHL12, TANGO1, and SAR1B. Surf4 interacts with apoB100 on VLDL and mediates the incorporation of VLDL into VTV. VLDL is then transported to the Golgi apparatus and secreted into the circulation from hepatocytes. In circulation, TG on VLDL is hydrolyzed by lipoprotein lipase (LPL) to IDL. TG on IDL can be further hydrolyzed by LPL and hepatic lipase (HL) to form LDL. Elevated LDL can be deposited in the arterial intima and then oxidized to ox-LDL, promoting the development of atherosclerosis. This figure is created with BioRender.com.

VLDL is a triglyceride (TG)-rich lipoprotein exclusively synthesized and secreted by hepatocytes for energy delivery to peripheral tissues, such as the heart and muscle. VLDL secretion is also one of the main means for hepatocytes to remove excessive TG. Lipoprotein lipase (LPL) hydrolyzes TG on VLDL, converting VLDL to intermediate-density lipoprotein (IDL). IDL can be further metabolized to LDL (Tiwari and Siddiqi, 2012; Ginsberg et al., 2021; Figure 3). Apolipoprotein B 100 (apoB100) is an essential structural protein of VLDL, IDL, and LDL and is only expressed in hepatocytes. Rodent hepatocytes express both apoB100 and apoB48, while apoB48 is only expressed in human enterocytes because human hepatocytes do not express APOBEC1, which edits apoB mRNA to generate apoB48 through converting a CAA codon at *apoB* mRNA to a UAA stop codon (Hirano et al., 1996; Nakamuta et al., 1996). VLDL biogenesis occurs in the ER lumen of hepatocytes, starting with the translocation of newly synthesized apoB100 through rough ER membrane. Nascent apoB100 is partially lipidated to form nascent VLDL particles cotranslationally. This step is mediated by microsomal triglyceride transport protein (MTP), a lipid transfer protein that transfers both neutral and polar lipids to newly synthesized apoB100. In the absence of sufficient lipids, unlipidated apoB100 is rapidly degraded mainly through the proteasome pathway (Tiwari and Siddiqi, 2012; Ginsberg et al., 2021). Nascent VLDL particles can acquire more lipids from ER lumen lipid droplets to generate VLDL. VLDL particles are then transported to the Golgi apparatus, where they undergo several modifications before being secreted into circulation. Inhibition of apoB100 or MTP markedly reduces VLDL secretion and plasma levels of LDL-C (Raabe et al., 1999; Conlon et al., 2016). Mipomersen (an antisense oligonucleotide targeting apoB100) and Lomitapide (a small inhibitor of MTP) are used to treat patients with homozygous familial hypercholesterinemia; however, both drugs cause severe side effects, such as hepatic lipid accumulation and liver damage (Santos et al., 2015; Blom et al., 2019). Mipomersen has been recently withdrawn from the market. Therefore, alternative targets to reduce VLDL secretion are urgently needed.

VLDL is a large particle up to 100 nm in diameter; therefore it cannot be transported via the classical COPII vesicles, which are only ∼60–70 nm in diameter (Barlowe and Helenius, 2016). Based on findings obtained from cryo-electron microscopy, Balch and colleagues reported that the geometry of COPII vesicles was flexible enough to form enlarged vesicles for cargoes up to ∼100 nm in diameter (Stagg et al., 2006). However, several lines of evidence show that VLDL does not exit the ER via classic COPII vesicles. Instead, VLDL departs the ER in a specialized vesicle, the VLDL transport vesicle (VTV) (Tiwari and Siddiqi, 2012; Santos et al., 2016; Tiwari et al., 2016). VTV ranges between 100 nm and 120 nm in diameter and can readily accommodate VLDL-sized cargos. VTV formation requires the COPII components, transport and Golgi organization 1 (TANGO1), SAR1B, and Kelch-like protein 12 (KLHL12) (Tiwari and Siddiqi, 2012; Butkinaree et al., 2014; Santos et al., 2016; Ginsberg, 2021; Figure 3). Silencing of KLHL12 significantly reduced secretion of apoB100 and resulted in accumulation of apoB in the ER of McArdle RH7777 cells, indicating its role in VLDL secretion (Butkinaree et al., 2014). KLHL12 is a key substrate adaptor protein for a Cul3-Ring ligase complex. Jin et al. (2012) reported that the KLHL12–Cul3 ubiquitin ligase mediates monoubiquitylation of SEC31, promoting the assembly of large COPII vesicles for procollagen secretion. It will be of interest to assess whether the same mechanism exists for KLHL12-mediated VLDL secretion. On the other hand, TANGO1 is a transmembrane protein resided in the ERES. Its N-terminal ER luminal SH3-like domain can bring bulky molecules, such as collagens, to the ERES, facilitating the ER export of these bulky cargos (Ishikawa et al., 2016; Santos et al., 2016; Rios-Barrera et al., 2017; Raote et al., 2018). Santos et al. (2016) reported that TANGO1 and TANGO1-like (TALI) protein were required for the formation of VTV and VLDL secretion in HepG2 cells. Depletion of TANGO1 impaired the ER export of apoB100 in HepG2 cells. However, whether and how the SH3-like domain of TANGO1 directly recognizes and then brings VLDL to the ERES remains elusive. In addition, several other proteins, such as TM6SF2 (Luo et al., 2022), Cideb (Ye et al., 2009; Tiwari et al., 2013), and SVIP (Tiwari et al., 2016), have been implicated in VLDL production. Surf4 has also been reported to directly interact with apoB100 and mediate apoB100 secretion from HepG2 cells (Saegusa et al., 2018). Knockdown of Surf4 in HepG2 cells significantly reduced secretion of apoB100 and resulted in accumulation of apoB100 inside the cells. Furthermore, they observed that Surf4 was localized in ERES, and Surf4 silencing reduced COPII-positive ERES in HepG2 cells, indicating its important role in maintaining ERES organization and protein export from the ER. Indeed, it has been reported that Surf4 facilitates secretion of various substrates, such as proinsulin (Saegusa et al., 2022), prosaposin (Devireddy and Ferguson, 2022), and EPO (Lin et al., 2020). On other hand, Mitrovic et al. (2008) reported that deficiency of Surf4 did not affect the total amount of proteins secreted from Hela cells even though Surf4, together with ERGIC-53 and p25, played an essential role in maintaining the architecture of ERGIC and Golgi. Therefore, Surf4 may mediate secretion of specific proteins in a cell type-dependent manner.

Recently, we and others demonstrated that Surf4 is required for VLDL secretion *in vivo* (Wang et al., 2021a, b; Shen et al., 2022). Knockout of hepatic Surf4 in mice significantly reduced TG secretion and plasma apoB, cholesterol, and TG levels. Inhibition of hepatic Surf4 also dramatically ameliorated the development of atherosclerosis in LDLR knockout mice fed the Western-type diet (Wang et al., 2021a). Consistent with LDLR knockout mice, knockdown of hepatic Surf4 in apolipoprotein E (apoE) knockout mice, another commonly used mouse model for studying atherosclerosis, significantly reduced atherosclerotic lesion areas (Shen et al., 2022). Furthermore, plasma apoA-I levels were dramatically reduced in Surf4 liver-specific knockout (Surf4^LKO^) mice. However, knockdown of Surf4 in cultured hepatocytes or mice significantly impaired secretion of apoB but not apoA-I, indicating that Surf4 is not essential for apoA-I secretion. Furthermore, Surf4^LKO^ mice did not show hepatic TG accumulation or notable liver damage in spite of impaired VLDL secretion. Taken together, these studies provide strong evidence that Surf4 mediates VLDL secretion and plays a critical role in regulating plasma lipoprotein homeostasis. However, further studies are needed to elucidate why impaired VLDL secretion does not cause liver steatosis in Surf4^LKO^ mice. The physiological impact of extremely low plasma cholesterol levels in Surf4^LKO^ mice, including LDL and HDL cholesterol, also needs to be assessed. For example, plasma lipoprotein cholesterol is the main substrate resource for the adrenal gland to produce steroid hormones. Indeed, Chang et al. (2021) observed a significant reduction in adrenal cholesterol levels in Surf4^LKO^ mice. However, the production of adrenal steroid hormones was comparable in the control and Surf4^LKO^ mice. They observed that the transcriptional activity of SREBP-2 and the expression of one of its target genes, 3-hydroxy-3-methylglutaryl-coenzyme A (HMG-CoA) reductase, were significantly increased in the adrenal gland of Surf4^LKO^ mice. HMG-CoA reductase is the rate-limiting enzyme in cholesterol biosynthesis, indicating an increase in cholesterol *de novo* biosynthesis. This may compensate for the loss of circulating lipoprotein-derived cholesterol in the adrenal gland of Surf4^LKO^ mice (Chang et al., 2021). Taken together, these findings suggest that hepatic Surf4 may be a promising target for lowering plasma levels of cholesterol, especially LDL-C, thereby reducing the risk of atherosclerotic cardiovascular disease with fewer side effects.

### Other physiological and pathophysiological functions of Surf4

Global Surf4 knockout (Surf4^−/−^) mice die as early as embryonic day 3.5 (E3.5) (Emmer et al., 2020). Knockout of apoB also results in embryonic lethality, but at a later stage (E9.5) (Farese et al., 1995). Therefore, the impact of Surf4 deficiency on embryonic development may not be related to its role in apoB secretion. Surf4 can promote cellular reprogramming and stimulate the generation of induced pluripotent stem cells (iPSCs). Its expression is high in metaphase II oocytes and early embryos prior to the two-cell stage (Wang et al., 2010; Gao et al., 2017; Wu et al., 2021). These findings indicate a critical role for Surf4 in embryonic development. Deficiency of Surf4 may lead to embryonic lethality due to embryonic dysplasia (Emmer et al., 2020; Wu et al., 2021). Alternatively, Surf4 may mediate secretion of unidentified cargoes that are important for early embryonic development.

Surf4 has also been shown to participate in the replication of positive-strand RNA viruses, such as hepatitis C virus (HCV) and poliovirus. Surf4 silencing significantly reduces virus replication but does not alter viral entry, translation, assembly, or release. Surf4 can be recruited into virus RNA replication complex via the HCV non-structural 4B protein, contributing to the formation of double-membrane vesicles (DMVs) that serves as a platform for viral replication (Kong et al., 2016, 2020; Snijder et al., 2020). Furthermore, Surf4 has been reported to have oncogenic potential in NIH3T3 cells. Surf4 is highly expressed in human cancer tissues, and patients with higher Surf4 levels have shorter overall survival. Consistently, overexpression of Surf4 increased cell proliferation and migration *in vitro*, and introduction of Surf4-overexpressing NIH3T3 cells into mice induced tumor growth (Kim et al., 2018). Surf4 expression is also upregulated in ovarian cancer stem cells, and knockdown of Surf4 inhibits tumorigenesis. How Surf4, a cargo receptor, possesses oncogenic potential remains elusive. Several recent studies have shed light on this question. Yue et al. (2020) used RNA-sequencing and bioinformatics to identify BIRC3 as a downstream regulator of Surf4. Surf4-knockdown human ovarian cancer cell lines, A2780 and 3AO, showed reduced protein and mRNA levels of BIRC3, suppressed self-renewal ability, and improved sensitivity to chemotherapeutic drugs. BIRC3 is an apoptosis inhibitor and functions through inhibiting caspase activation (Frazzi, 2021). In addition, Tang et al. (2022) demonstrated that Surf4 was required for sonic hedgehog (Shh) export from the ER in Hela and HEK293T cells. Shh is an important signaling molecule that plays a critical role in cell differentiation and contributes to the development and progression of numerous cancers (Wang et al., 2022). Therefore, Surf4 may exert its oncogenic role by upregulating BIRC3 expression and/or promoting ER–Golgi trafficking of Shh.

## Conclusions and perspective

Surf4 has been identified as an ER cargo receptor 10 years ago. It can recognize and mediate ER export of a wide variety of cargos and plays a complex role in various physiological and pathophysiological processes, such as mediating VLDL secretion, promoting oncogenesis, facilitating virus replication, and regulating embryonic development. These findings indicate that Surf4 is a promising therapeutic target, and more studies are needed to dissect Surf4’s function and underlying mechanisms thoroughly. (i) Surf4 recognizes and mediates export of diverse cargos. Does it contain distinct binding sites for specific cargos? (ii) The ER-ESCAPE motif in cargos is required for Surf4-mediated ER export of some secretory proteins. However, Surf4 also facilitates ER export of cargos without the motif. Do these cargos bear unidentified specific amino acid sequences for Surf4 recognition and sorting? (iii) Given the diversity of cargos exported by Surf4, does the cargo receptor require additional control factors to ensure cargo selectivity? (iv) Does Surf4 mediate secretion of specific cargos in a cell type-dependent manner? For example, Surf4 can facilitate secretion of PCSK9 from HEK293 cells and cardiomyocytes but not hepatocytes.

## Notes

**Author contributions:** Y.S. wrote the initial draft. S.Q. and D.-W.Z. supervised the final version. D.-W.Z. wrote the final version. H.-M.G. participated in the discussion and the preparation of the manuscript.
